# ﻿*Halamphorahampyeongensis* sp. nov. (Amphipleuraceae, Bacillariophyceae), a new marine benthic diatom from a tidal mudflat in Hampyeong Bay, South Korea

**DOI:** 10.3897/phytokeys.248.135034

**Published:** 2024-10-22

**Authors:** Sung Min An, Jihoon Kim, Kichul Cho, Hyun-Ju Hwang

**Affiliations:** 1 Department of Biological Application & Technology, National Marine Biodiversity Institute of Korea, Seocheon 33662, Republic of Korea National Marine Biodiversity Institute of Korea Seocheon Republic of Korea; 2 LMO Research Team, Bureau of Ecological Research, National Institute of Ecology, Seocheon 33657, Republic of Korea National Institute of Ecology Seocheon Republic of Korea

**Keywords:** 18S, morphology, new species, phylogeny, *rbc*L gene, taxonomy

## Abstract

The abundance and variety of benthic diatoms inhabiting tidal flats is widely acknowledged, although it has received relatively less attention than other research areas. In this investigation, we provide a formal description of a benthic diatom found in the tidal mudflat of South Korea, based on morphological and molecular characteristics and the similarities and differences between *Halamphorahampyeongensis***sp. nov.**, with morphologically similar *Halamphora* species are also discussed. Morphological characteristics are described from light and electron microscopy images. *H.hampyeongensis* is distinguished by its wide ventral sides of the valve, small and rounded areolae present across the whole valve face, and dense dorsal striae biseriate (34–38 in 10 μm). Phylogenetic analysis based on 18S rDNA and *rbc*L sequence data revealed that *H.hampyeongensis* is related to *H.montana*, *H.mosensis*, and *H.specensa*. The results (morphometric and molecular) provide sufficient elements to support and propose this taxon as a new species.

## ﻿Introduction

Amphoroid diatoms, represented among other genus by *Amphora* Ehrenberg ex Kützing 1844 and *Halamphora* (Cleve) Mereschkowsky, 1903, are strongly dorsiventral raphid diatoms (Vyverman et al. 1998). The classification of the genus *Amphora* has been a subject of extensive study due to its non-monophyly (Cleve 1895; Mereschkowsky 1903; Krammer 1980; Mann 1994; Edlund et al. 2009) and prompting numerous attempts to accurately categorize this large genus (Cleve 1895; Cleve-Euler 1953; Levkov 2009; Stepanek and Kociolek 2016). Particularly, Cleve (1895), based on frustule morphology, erected nine subgenera included *Halamphora*. His proposal emerged as the most widely recognized classification system for *Amphora*. Moreover, Mereschkowsky (1903) proposed a new division of the genus *Amphora* into four genera (*Clevamphora*, *Cymbamphora*, *Halamphora*, and *Tetramphora*) based on the number and morphology of the plastids in the cells. Among them, *Halamphora* contains no libroplasts, which are highly refractile, approximately spherical volutin granules (Mann 1989); however, this classification has not been widely accepted (Stepanek and Kociolek 2014). Levkov (2009) proposed moving *Halamphora* from the subgenus level to the genus level by transferring 59 existing species of the genus *Amphora* and adding 16 new species to it. The genus was established on the basis of the distinctive H-shaped single plastids, which are appressed ventrally and exhibit longitudinal constriction in the central region of the H-shape (Levkov 2009). This morphological feature aligns with the second type of plastid arrangement as proposed by Mereschkowsky (1903). The most obvious morphological feature that distinguishes the genus *Halamphora* from the genus *Amphora* is the absence of raphe ledge on the ventral side of the valves in the genus *Halamphora* (Ács et al. 2011). In addition, the genus *Halamphora* has the following morphological characteristics: striae composed of areolae with recessed foramina; internal central raphe endings terminating onto a fused central helictoglossae (Levkov 2009; Ács et al. 2011). Although a comprehensive and systematic revision of *Amphora* is currently underway, numerous molecular phylogenetic studies provide strong evidence supporting the monophyletic status of *Halamphora* as a distinct group separate from *Amphorasensu stricto* (Ruck and Theriot 2011; Sato et al. 2013; Stepanek and Kociolek 2014; Stepanek and Kociolek 2019; An et al. 2022).

Since its recognition as a genus by Levkov (2009), there has been a growing understanding of the diversity within the genus *Halamphora*. Furthermore, there has been a significant increase in the determination of new species belonging to this genus (Álvarez-Blanco and Blanco 2014; Jiang et al. 2015; Olivares-Rubio et al. 2017; López-Fuerte and Siqueiros-Beltrones 2018; Stepanek and Kociolek 2018; Zhang et al. 2019; López-Fuerte et al. 2020; An et al. 2022). According to AlgaeBase, the genus *Halamphora* constitute 155 accepted species names, including eight varieties, and is currently classified in the family Amphipleuraceae (Guiry and Guiry 2024). However, Torres-Ariño et al. (2019) deemed it appropriate to classify the species in the genus *Halamphora* under the family Catenulaceae instead of Amphipleuraceae due to the presence of dorsal marginal thickening and the absence of dorsal fascia. This is also confirmed by the phylogenetic tree of Olivares-Rubio et al. (2017).

Ecologically, *Halamphora* species are known to prefer mostly inland conductive waters or coastal waters (Levkov 2009; Stepanek and Kociolek 2018; Sala et al. 2021; Spaulding et al. 2021). Tidal flats have also been recognized as a significant habitat for the genus *Halamphora* (Desianti et al. 2017; Plante et al. 2021). However, the genus *Halamphora*, including the broader *Amphorasensu lato* group, is fairly understudied in tidal flats (An et al. 2020).

In the present study, we used light microscopy and scanning electron microscopy (SEM) to conduct morphological examinations on a novel *Halamphora* species that was isolated from a tidal mudflat in Hampyeong Bay, South Korea. We additionally performed molecular analysis of this species using 18S rDNA and *rbc*L gene, and a brief discussion has been included regarding these findings.

## ﻿Materials and methods

### ﻿Study site, isolation and cultivation

**Figure 1.**
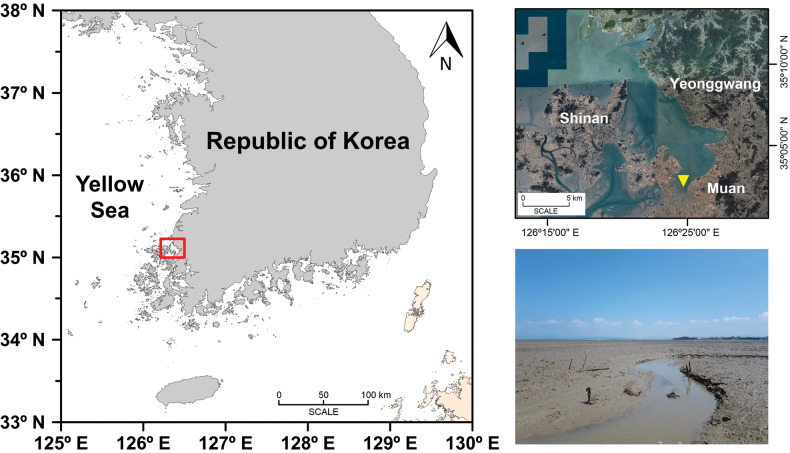
Location of the sampling site in Hampyeong Bay at the west coast of Korea. The satellite image included in this figure was sourced from Google Earth.

The Sediment sample was obtained from an intertidal mudflat located in Hampyeong Bay (35º01.89'N, 126º24.31'E) on the west coast of South Korea on July 19, 2018 (Fig. 1). To obtain sediment sample containing diatoms, the surface of the tidal flat was scratched to a depth of ca. 2 mm by spatula and the sediment collected in a 50 mL conical tube. Hampyeong Bay is characterized as a semi-closed bay, encompassing a vast tidal flat spanning approximately 4,700 hectares. The bay does not receive any significant inflow from large rivers, and the contribution of fresh water from small streams is negligible. The annual mean values of temperature and salinity of pore waters in sediments varied between 12.7 and 28.6 °C and between 9.5 and 25 psu, respectively (Hwang and Koh 2012). The sediment temperature and salinity of pore water were measured using a thermometer equipped with a stainless-steel probe (Daihan Scientific Co., Wonju, South Korea) and a YSI Pro 1030 multi-parameter instrument (YSI, Yellow Springs, OH, USA), respectively. At the time of sampling, the sediment temperature was recorded at 27.8 °C, while the salinity of the pore water was measured at 24.9 psu.

A single cell was isolated using a capillary tube under an Eclipse Ti-U inverted microscope (Nikon, Tokyo, Japan) and transferred to a cell culture flask (SPL Life Sciences, Pocheon, South Korea) containing F/2 medium supplemented with silicate (Sigma Aldrich, St. Louis, MO, USA). The strains were periodically sub-cultured every 3–4 weeks and subjected to incubation at a temperature of 25 °C. The incubation was carried out under a light:dark cycle of 14:10 hours, with an irradiance of 40 μmol photons m^-2^ s^-1^.

### ﻿Morphological observations

For the purpose of conducting morphological analysis, a sample of the culture was obtained from the initial subculture and treated with a 5% Lugol’s solution for fixation. The cultured strain was treated acid treatment using sulfuric and hydrochloric acids to remove organic matter, following the modified Hendey (1974). Subsequently, the strain was affixed onto permanent slides using Pleurax (Wako Pure Chemical Industries, Osaka, Japan). Three slides were analyzed utilizing an AX10 light microscope (LM) that was equipped with an Imager A2 digital camera system (Zeiss, Göttingen, Germany). The examination was conducted under a 100× Plan-Apochromat oil-immersion objective lens (N.A. 1.30). SEM analysis was conducted following the protocol outlined by An et al. (2022). The cleaned specimens were subjected to a filtration process using a polycarbonate membrane (25 mm in diameter with a pore size of 2 μm). This was rinsed with distilled water. The membrane underwent dehydration through a series of graded ethanol concentrations ranging from 10 to 100%. Subsequently, it was dried using tetramethylsilane (Sigma Aldrich). The membrane was affixed to an aluminium stub and subjected to gold sputter-coating using an MC1000 ion sputter (Hitachi, Tokyo, Japan). The stub was used for examination utilizing a Sigma 500-VP high-resolution field-emission scanning electron microscope (Zeiss).

### ﻿Phylogenetic analysis

The 1.5 mL of culture strain was collected through centrifugation at a speed of 2,500 rpm for a duration of 5 minutes, and the supernatant was discarded. Genomic DNA extraction was performed using the DNeasy PowerSoil Pro Kit (Qiagen Inc., Hilden, Germany) according to the manufacturer’s instructions. Polymerase chain reaction (PCR) amplification was conducted using specific primer pairs. The primer pair Diatom9F (Lynch et al. 1997) and EukBR (Medlin et al. 1988) were used to amplify the 18S rDNA region. Additionally, the primer pair DPrbcL1 / DPrbcL7 (Daugbjerg and Andersen 1997) was employed to amplify the *rbc*L gene.

PCR conditions and reactions were implemented as per the protocols of (An et al. 2017). The PCR product was purified using the ExoSAP-IT Express PCR Product Cleanup Reagent (Thermo Fisher Scientific, Waltham, MA, USA) and was subsequently sequenced by Macrogen Inc. (Seoul, South Korea). Sequence was trimmed, assembled and aligned using Geneious Prime v.2023.0.1 (Biomatters Ltd., Auckland, New Zealand). Phylogenetic trees were constructed using the Randomized Axelerated Maximum Likelihood (RAxML) v.8.2.10 (Stamatakis 2014) and MrBayes version 3.2.7 (Ronquist and Huelsenbeck 2003), based on maximum likelihood (ML) and Bayesian phylogenetic inference (BI) methods. ML analysis was conducted utilizing the GTRGAMMAIX model selected by ModelTest-NG v.0.1.7 (Darriba et al. 2020), with the number of bootstrap replicates set to 1,000 and all other settings kept at their default values. BI was conducted using the established methods as outlined in the study by López-Fuerte et al. (2020). A dataset of concatenated 18S rDNA and *rbc*L sequence data was constructed, including 80 Amphoroid diatom sequences from GenBank (Suppl. material 1). The dataset exclusively includes information on strains for which both 18S and *rbc*L sequences are available. The outgroup for this analysis was *Tetramphorachilensis* (Hustedt) Stepanek & Kociolek, 2016 strain 8531-Amph132. Trees were visualized using Figtree v. 1.4.4 and Adobe Illustrator v. 27.1.1 (Adobe Systems, San José, CA, USA).

## ﻿Results

### ﻿ Halamphora hampyeongensis

Taxon classificationPlantaeNaviculalesAmphipleuraceae

S.M.An & J.Kim sp. nov.

4D988655-00B8-575C-BFEF-5DC3C787298A

Fig. 2

#### Description.

**Figure 2.**
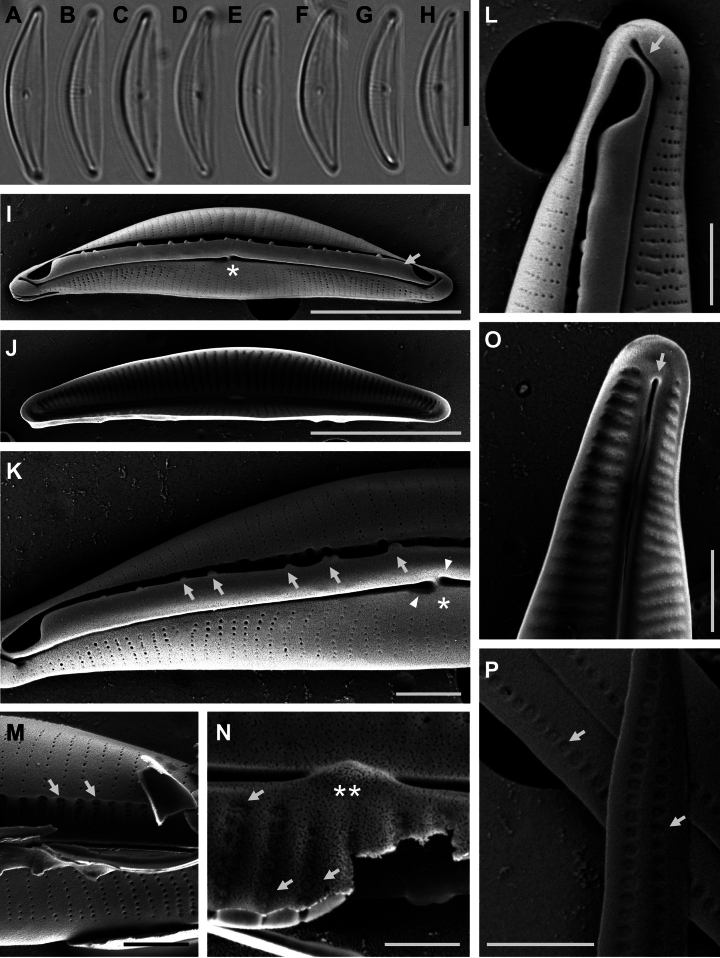
**A–H** Light microscopy photomicrographs of *H.hampyeongensis***I–P** scanning electron microscopy photomicrographs of *H.hampyeongensis***I** external valve view, with central area (asterisk) and dorsal raphe ledge (arrow) **J** internal view of a valve **K** detail of a valve externally showing siliceous outgrowths (arrows) on the margin of the raphe ledge, central area (asterisk), and proximal raphe endings (arrowheads) **L** detail of external valve apex showing the dorsally bent distal raphe ending (arrow) **M** biseriate striae (arrows) in several rows under the raphe ledge **N** detail of areolae on the dorsal side internally occluded by hymenes (arrows) and tongue-like proximal helictoglossae (double asterisk) **O** detail of internal valve apex showing poorly developed distal helictoglossae (arrow) **P** girdle bands with two rows of poroids (arrows). Scale bars: 10 μm (**A–H**); 5 μm (**I**, **J**); 1 μm (**K–M**, **O**, **P**); 0.5 μm (**N**).

In LM, valves semi-elliptical with smoothly convex dorsal margin, nearly straight ventral margin and the valve ends narrowly rounded and slightly ventrally curved (Fig. 2A–H). Raphe slightly arched and positioned centrally to slightly ventrally on the valve face. Both dorsal and ventral striae not discernible. Valve length 13.8–15.0 μm; valve breadth 2.8–3.0 μm (n = 31).

In SEM, externally, the central area slightly expanded on the ventral side only (Fig. 2I, K, asterisks). Central raphe endings straight, slightly expanded and positioned very close together (Fig. 2K, arrowhead); distal raphe endings bent towards the dorsal side (Fig. 2L, arrow). Raphe ledge well-developed on the dorsal side of the valve (Fig. 2I, K) and narrows sharply towards the dorsal valve margin. Siliceous outgrowths of various sizes arranged irregularly on the raphe ledge margin (Fig. 2K, arrows). The axial area narrow, and difficult to distinguish because of the fine striae (Fig. 2I).

Dorsal striae slightly radiate. Ventral striae are slightly radiate and more distantly spaced at the valve middle, becoming parallel to slightly convergent and denser near the valve apices (Fig. 2I, K, M). Dorsal striae biseriate under raphe ledge with two rows of small, round areolae under SEM (Fig. 2M, arrow); becoming uniseriate, composed of very small round areolae over the rest of the dorsal side (Fig. 2K, M), 34–38 in 10 μm (n = 13). Ventral striae uniseriate, almost identical in size and shape to dorsal striae, 46–48 in 10 μm (n = 13, Table 1). Axial longitudinal line absent on dorsal side. Internally, longitudinal rib absent. Areolae occluded by hymenes (Fig. 2N, arrows). Proximal raphe endings finish onto small and tongue-shaped fused helictoglossae (Fig. 2N, double asterisk). Poorly developed helictoglossae at distal raphe endings (Fig. 2O, arrow). Round or ovoid poroids in girdle bands, arranged in two rows of 65–67 in 10 μm (Fig. 2P, arrows).

**Table 1.** Morphometric comparation of *Halamphorahampyeongensis* with related species. ND = not documented.

	*Halamphorahampyeongensis*	*H.atacamana*	*H.caribaea*	*H.exilis*	*H.montana*	*H.mosensis*	*H.specensa*
Valve shape	semi-elliptical, convex dorsal and nearly straight ventral margins	semi-lanceolate, arched dorsal and straight to weakly tumid ventral margins	semi-lanceolate, convex dorsal and straight ventral margins	narrowly semi-elliptical, shallowly arched dorsal and straight ventral margins	semi-lanceolate, smoothly arched dorsal and straight to slightly convex ventral margins	narrowly semi-elliptical, smoothly arched dorsal and straight ventral margins	semi-elliptical to nearly elliptical, arched to flattened dorsal and convex ventral margins
Apices	narrowly rounded	slightly subprotracted	rostrate	narrowly rounded	broadly rounded	narrowly rounded	weakly protracted, narrowly rounded
Length (μm)	13.8–15.0	29–45	34–39	14–19	12–20	21–37	13–17
Width (μm)	2.8–3.0	4.5–8.0	6.0–8.0	2.5–3.0	3.0–4.6	4.0–4.5	3.0–3.5
Raphe	arched	arched	straight with	slightly arched	arched	arched	straight
Proximal raphe ends	slightly expanded, straight	slightly dorsally deflected	dorsally deflected	slightly dorsally deflected	slightly expanded, dorsally deflected	dorsally deflected	dorsally deflected
Dorsal striae (in 10 μm)	34–38	25–28	11–20	24–26	40–45	26–28	23–26
	bi- and uniseriate	uniseriate	ND	bi- and uniseriate	uniseriate	bi- and multiseriate	biseriate
Ventral striae (in 10 μm)	46–48	24–30	19–29	44–45	40–45	28	44–46
Habitate	brackish	brackish	marine	marine	freshwater	brackish	freshwater
References	This study (n = 31)	Levkov (2009)	Wachnicka and Gaiser (2007)	Stepanek and Kociolek (2018)	Watanabe et al. (2005); Levkov (2009)	Stepanek and Kociolek (2018)	Stepanek and Kociolek (2018)

#### Holotype.

Slide no. MABIK DI00043482 (represented by the valve shown in Fig. 2A) was deposited at the National Marine Biodiversity Institute of Korea (MABIK), located in Seocheon-gun, Chungcheongnam-do, South Korea.

#### Isotype.

SEM stub no. MABIK DI00043483 and cleaned material no. MABIK DI00043484 (preserved in 99% ethanol).

#### Type locality.

The intertidal mudflat located in Hampyeong Bay (35°01.89'N, 126°24.31'E), Muan-gun, Jeollanam-do, South Korea (site: HP1-3), July 19, 2018.

#### Etymology.

The specific epithet “*hampyeongensis*” refers to the type locality, Hampyeong Bay, Muan-gun, Jeollanam-do, South Korea.

#### Distribution and ecology.

*Halamphorahampyeongensis* is a benthic species currently known only from the type locality. The sediment temperature and salinity of pore water in the sediment at the time of sampling were measured to be 27.8 °C and 24.9 psu, respectively.

#### Gene sequences.

The nucleotide sequences were deposited in GenBank under accession numbers OQ642108 (18S rDNA) and ON137728 (*rbc*L gene).

##### ﻿Phylogenetic analysis

**Figure 3.**
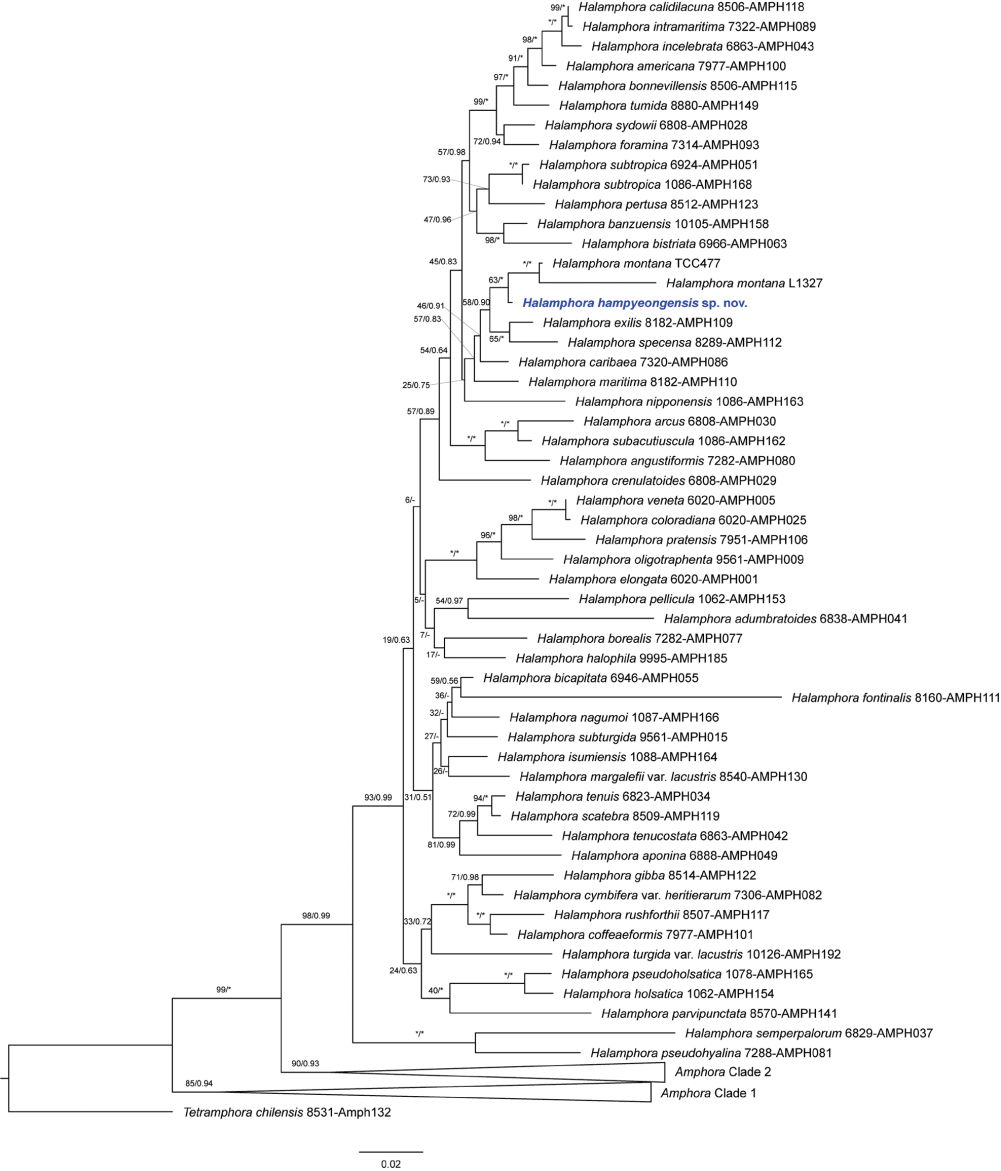
A molecular phylogenetic tree obtained from ML and BI analysis based on the concatenated dataset (18S rDNA and *rbc*L gene) showing the phylogenetic positions of *H.hampyeongensis*. A phylogenetic tree was generated through a ML analysis. The values on each node indicate ML bootstrap and Bayesian posterior probabilities, respectively. The asterisk indicates 100 in ML and 1 in BI, and – indicates the topological incongruence between ML and BI trees. The *H.hampyeongensis* is highlighted in bold blue.

The lengths of the 18S rDNA and *rbc*L gene sequences were determined in this study for *Halamphorahampyeongensis* were 1,649 bp and 1,424 bp, respectively. Phylogenetic analysis was conducted to determine the relationship between *H.hampyeongensis* and the amphoroid diatom species retrieved from GenBank using ML and BI methods (Fig. 3). The topologies of the phylogenetic trees were similar regardless of the phylogenetic analysis methods and molecular regions utilized. Phylogenetic analysis provided robust support for the monophyletic grouping of *Halamphora* species including *H.hampyeongensis*, as indicated by a maximum likelihood bootstrap support of 98% and a Bayesian posterior probability of 0.99. *H.hampyeongensis* was located in *Halamphora* Clade Hal_H as described by Stepanek and Kociolek (2019) with a low bootstrap value (bootstrap value = 25). This species also showed weak support (bootstrap value = 63) as the sister taxon to *H.montana* (Krasske) Levkov.

## ﻿Discussion

*Halamphorahampyeongensis* sp. nov. displayed typical morphological characteristics associated with the Amphoroid as observed through microscopic examination (Fig. 2). Furthermore, phylogenetic analysis indicates that *H.hampyeongensis* belongs to the clade that includes *Halamphora* strains (Fig. 3). Therefore, considering its morphological features and molecular data, the classification of *H.hampyeongensis* in the genus *Halamphora* is deemed appropriate.

As a result of phylogenetic analysis, this species was found to be related to *Halamphora* Clade Hal_H, which consists of species like *H.exilis* J.G.Stepanek & Kociolek, *H.mosensis* J.G.Stepanek & Kociolek, *H.specensa* J.G.Stepanek & Kociolek, *H.maritima* J.G.Stepanek & Kociolek and *H.nipponensis* J.G.Stepanek, S.Mayama & Kociolek as described by Stepanek and Kociolek (2019). This clade is characterized by specific morphological traits, including the absence of a dorsal marginal ridge, the presence of biseriate internal areolae occlusions, and hymen internal areolae occlusions. Notably, H.maritima and H.nipponensis exhibit distinct differences from the other species within the clade, specifically in their narrow ventral valve and ventral striae, which are composed of elongated areolae. In contrast, the other species typically possess a relatively wide ventral valve and fine uniseriate ventral striae. These characteristics are also observed in the *H.hampyeongensis* sp. nov. *H.hampyeongensis* shares similarities with *H.caribaea* (Wachnicka & E.E.Gaiser) Rimet & R.Jahn, *H.exilis*, *H.mosensis*, and *H.specensa* in overall valve outline, relatively broad ventral side of the valves, and specific striae characteristics, including fine uniseriate ventral striae and biseriate dorsal striae near the axial area. *H.hampyeongensis* has smaller areolae and denser striae (34–38 in 10 μm) compared to *H.caribaea* (11–20 in 10 μm) and *H.exilis* (24–26 in 10 μm) (Table 1) (Wachnicka and Gaiser 2007; Stepanek and Kociolek 2018). Unlike *H.hampyeongensis*, *H.mosensis* has multi-seriate dorsal striae, and *H.specensa* has a convex ventral margin and irregularly bi-seriate dorsal striae (Stepanek and Kociolek 2018). In addition, *H.hampyeongensis* shares fine areolae with *H.atacamana* (Patrick) Levkov, and *H.montana*. However, *H.atacamana* can be differentiated from *H.hampyeongensis* based on its valve outline and lower stria density (25–28 in 10 μm) (Levkov 2009), and *H.montana* possesses semi-stauros, making it easily distinguishable from *H.hampyeongensis*, even when observed under a light microscope (Watanabe et al. 2005; Levkov 2009). Furthermore, this species exhibits distinctive ornamentation on its raphe ledge, which differentiates it from other *Halamphora* species (Fig. 2K, arrows). Nevertheless, as this observation has not been substantiated through natural samples, additional verification is necessary.

Based on the aforementioned information, we propose a novel taxon found in the mudflats as a new species belonging to the genus *Halamphora*, named *H.hampyeongensis*. While benthic diatoms are the predominant organisms in benthic ecosystems of tidal flats and are recognized for their high diversity (Underwood and Barnett 2006), their overall understanding is still limited. In the future, further research is required to elucidate the species richness of diatoms in tidal flats. Consequently, it is anticipated that new and previously undocumented species will continue to be identified and reported.

## Supplementary Material

XML Treatment for Halamphora hampyeongensis

## ﻿Additional information

### Author ORCIDs

Sung Min An https://orcid.org/0000-0002-9180-309X

Jihoon Kim https://orcid.org/0000-0001-6363-6514

Kichul Cho https://orcid.org/0000-0002-0685-0490

Hyun-Ju Hwang https://orcid.org/0009-0008-4608-7021

### Data availability

All of the data that support the findings of this study are available in the main text or Supplementary Information.

## Supplementary materials

Supplementary material 1 GenBank accession numbers for the amphoroid species included in the phylogenetic analyses Data type docx https://binary.pensoft.net/file/1158281This dataset is made available under the Open Database License (http://opendatacommons.org/licenses/odbl/1.0/). The Open Database License (ODbL) is a license agreement intended to allow users to freely share, modify, and use this Dataset while maintaining this same freedom for others, provided that the original source and author(s) are credited.Sung Min An, Jihoon Kim, Kichul Cho, Hyun-Ju Hwang

Supplementary material 2 Supplementary data Data type fas https://binary.pensoft.net/file/1158282This dataset is made available under the Open Database License (http://opendatacommons.org/licenses/odbl/1.0/). The Open Database License (ODbL) is a license agreement intended to allow users to freely share, modify, and use this Dataset while maintaining this same freedom for others, provided that the original source and author(s) are credited.Sung Min An, Jihoon Kim, Kichul Cho, Hyun-Ju Hwang
