# Supplementary material for: ﻿Halamphorahampyeongensis sp. nov. (Amphipleuraceae, Bacillariophyceae), a new marine benthic diatom from a tidal mudflat in Hampyeong Bay, South Korea
Source: PhytoKeys. 2024 Oct 22;248:59–71. doi: 10.3897/phytokeys.248.135034 (PMC11522744; doi:10.3897/phytokeys.248.135034)
Supplement: Supplementary material 1 — GenBank accession numbers for the amphoroid species included in the phylogenetic analyses [file phytokeys-248-059_article-135034__-s001.docx]

**Table S1.** GenBank accession numbers for the amphoroid species included in the phylogenetic analyses.

| Species name | Collection | GenBank accession no. | | Reference |
| --- | --- | --- | --- | --- |
|  |  | 18S rDNA | *rbc*L gene |  |
| *Amphora abludens* | 6961-AMPH059 | KJ463425 | KJ463455 | Stepanek & Kociolek (2014) |
| *Amphora affinis* | 9556-AMPH016 | KJ463424 | KJ463454 | Stepanek & Kociolek (2014) |
| *Amphora aliformis* | 10105-AMPH159 | MG027323 | MG027489 | Stepanek & Kociolek (2019) |
| *Amphora allanta* | 8539-AMPH129 | MG027314 | MG027479 | Stepanek & Kociolek (2019) |
| *Amphora beaufortiana* | 7278-AMPH074 | KJ463427 | KJ463457 | Stepanek & Kociolek (2014) |
| *Amphora calumetica* | 0611-AMPH094 | MG027290 | MG027455 | Stepanek & Kociolek (2019) |
| *Amphora commutata* | L1060 | KX120667 | KX120550 | Ruck et al. (2016) |
| *Amphora copulata* | 0778-AMPH095 | MG027291 | MG027456 | Stepanek & Kociolek (2019) |
| *Amphora gigantea* var. *fusca* | 6961-AMPH099 | MG027294 | MG027459 | Stepanek & Kociolek (2019) |
| *Amphora graeffeana* | 6925-AMPH069 | MG027282 | MG027447 | Stepanek & Kociolek (2019) |
| *Amphora helenensis* | SZCZCH704 | KT943649 | KT943672 | Witkowski et al. (2016) |
| *Amphora indistincta* | 9559-AMPH020 | KJ463433 | KJ463463 | Stepanek & Kociolek (2014) |
| *Amphora laevissima* | 7314-AMPH085 | KJ463434 | KJ463464 | Stepanek & Kociolek (2014) |
| *Amphora lativentralis* | 6821-AMPH033 | MG027268 | MG027432 | Stepanek & Kociolek (2019) |
| *Amphora libyca* | AT-117.10 | AM501959 | AM710425 | Bruder & Medlin (2007) |
| *Amphora obtusa* var. *crassa* | 6951-AMPH070 | KJ463436 | KJ463466 | Stepanek & Kociolek (2014) |
| *Amphora ovalis* | 9490-AMPH013 | KJ463437 | KJ463467 | Stepanek & Kociolek (2014) |
| *Amphora pediculus* | L1030 | HQ912417 | HQ912403 | Ruck & Theriot (2011) |
| *Amphora polita* | 1082-AMPH170 | MG027332 | MG027498 | Stepanek & Kociolek (2019) |
| *Amphora proteoides* | 7278-AMPH096 | MG027292 | MG027457 | Stepanek & Kociolek (2019) |
| *Amphora proteus* | 6961-AMPH071 | KJ463439 | KJ463469 | Stepanek & Kociolek (2014) |
| *Amphora sublaevis* | 6829-AMPH038 | KJ463443 | KJ463473 | Stepanek & Kociolek (2014) |
| *Amphora vixvisibilis* | SZCZCH967 | KT943648 | KT943670 | Witkowski et al. (2016) |
| *Amphora waldeniana* | 8348-AMPH011 | KJ463447 | KJ463477 | Stepanek & Kociolek (2014) |
| *Halamphora adumbratoides* | 6838-AMPH041 | MG027270 | MG027434 | Stepanek & Kociolek (2019) |
| *Halamphora americana* | 7977-AMPH100 | MG027295 | MG027460 | Stepanek & Kociolek (2019) |
| *Halamphora angustiformis* | 7282-AMPH080 | MG027285 | MG027450 | Stepanek & Kociolek (2019) |
| *Halamphora aponina* | 6888-AMPH049 | MG027275 | MG027439 | Stepanek & Kociolek (2019) |
| *Halamphora arcus* | 6808-AMPH030 | MG027267 | MG027431 | Stepanek & Kociolek (2019) |
| *Halamphora banzuensis* | 10105-AMPH158 | MG027322 | MG027488 | Stepanek & Kociolek (2019) |
| *Halamphora bicapitata* | 6946-AMPH055 | MG027278 | MG027442 | Stepanek & Kociolek (2019) |
| *Halamphora bistriata* | 6966-AMPH063 | MG027280 | MG027444 | Stepanek & Kociolek (2019) |
| *Halamphora bonnevillensis* | 8506-AMPH115 | MG027305 | MG027470 | Stepanek & Kociolek (2019) |
| *Halamphora borealis* | 7282-AMPH077 | MG027283 | MG027448 | Stepanek & Kociolek (2019) |
| *Halamphora calidilacuna* | 8506-AMPH118 | MG027307 | MG027472 | Stepanek & Kociolek (2019) |
| *Halamphora caribaea* | 7320-AMPH086 | KJ463428 | KJ463458 | Stepanek & Kociolek (2014) |
| *Halamphora coffeiformis* | 7977-AMPH101 | KJ463449 | KJ463479 | Stepanek & Kociolek (2014) |
| *Halamphora coloradiana* | 6020-AMPH025 | KJ463450 | KJ463480 | Stepanek & Kociolek (2014) |
| *Halamphora crenulatoides* | 6808-AMPH029 | MG027266 | MG027430 | Stepanek & Kociolek (2019) |
| *Halamphora cymbifera* var. *heritierarum* | 7306-AMPH082 | MG027287 | MG027452 | Stepanek & Kociolek (2019) |
| *Halamphora elongata* | 6020-AMPH001 | MG027259 | MG027423 | Stepanek & Kociolek (2019) |
| *Halamphora exilis* | 8182-AMPH109 | MG027301 | MG027466 | Stepanek & Kociolek (2019) |
| *Halamphora fontinalis* | 8160-AMPH111 | MG027303 | MG027468 | Stepanek & Kociolek (2019) |
| *Halamphora foramina* | 7314-AMPH093 | MG027289 | MG027454 | Stepanek & Kociolek (2019) |
| *Halamphora gibba* | 8514-AMPH122 | MG027310 | MG027475 | Stepanek & Kociolek (2019) |
| *Halamphora halophila* | 9995-AMPH185 | MG027335 | MG027501 | Stepanek & Kociolek (2019) |
| *Halamphora holsatica* | 1062-AMPH154 | MG027321 | MG027487 | Stepanek & Kociolek (2019) |
| *Halamphora hyalina* | 6963-AMPH061 | KJ463431 | KJ463461 | Stepanek & Kociolek (2014) |
| *Halamphora incelebrata* | 6863-AMPH043 | MG027272 | MG027436 | Stepanek & Kociolek (2019) |
| *Halamphora intramaritima* | 7322-AMPH089 | MG027288 | MG027453 | Stepanek & Kociolek (2019) |
| *Halamphora isumiensis* | 1088-AMPH164 | MG027326 | MG027492 | Stepanek & Kociolek (2019) |
| *Halamphora margalefii* var. *lacustris* | 8540-AMPH130 | MG027315 | MG027480 | Stepanek & Kociolek (2019) |
| *Halamphora maritima* | 8182-AMPH110 | MG027302 | MG027467 | Stepanek & Kociolek (2019) |
| *Halamphora montana* | L1327 | KX120666 | KX120549 | Ruck et al. (2016) |
| *Halamphora montana* | TCC477 | KC736615 | KC736590 | Kermarrec et al. (2013) |
| *Halamphora nagumoi* | 1087-AMPH166 | MG027328 | MG027494 | Stepanek & Kociolek (2019) |
| *Halamphora nipponensis* | 1086-AMPH163 | MG027325 | MG027491 | Stepanek & Kociolek (2019) |
| *Halamphora oligotraphenta* | 9561-AMPH009 | KJ463451 | KJ463481 | Stepanek & Kociolek (2014) |
| *Halamphora parvipunctata* | 8570-AMPH141 | MG027318 | MG027483 | Stepanek & Kociolek (2019) |
| *Halamphora specensa* | 8289-AMPH112 | MG027304 | MG027469 | Stepanek & Kociolek (2019) |
| *Halamphora pellicula* | 1062-AMPH153 | MG027320 | MG027486 | Stepanek & Kociolek (2019) |
| *Halamphora pertusa* | 8512-AMPH123 | MG027311 | MG027476 | Stepanek & Kociolek (2019) |
| *Halamphora pratensis* | 7951-AMPH106 | MG027299 | MG027464 | Stepanek & Kociolek (2019) |
| *Halamphora pseudoholsatica* | 1078-AMPH165 | MG027327 | MG027493 | Stepanek & Kociolek (2019) |
| *Halamphora pseudohyalina* | 7288-AMPH081 | MG027286 | MG027451 | Stepanek & Kociolek (2019) |
| *Halamphora rushforthii* | 8507-AMPH117 | MG027306 | MG027471 | Stepanek & Kociolek (2019) |
| *Halamphora scatebra* | 8509-AMPH119 | MG027308 | MG027473 | Stepanek & Kociolek (2019) |
| *Halamphora semperpalorum* | 6829-AMPH037 | KJ463441 | KJ463471 | Stepanek & Kociolek (2014) |
| *Halamphora subacutiuscula* | 1086-AMPH162 | MG027324 | MG027490 | Stepanek & Kociolek (2019) |
| *Halamphora subtropica* | 6924-AMPH051 | KJ463445 | KJ463475 | Stepanek & Kociolek (2014) |
| *Halamphora subtropica* | 1086-AMPH168 | MG027330 | MG027496 | Stepanek & Kociolek (2019) |
| *Halamphora subturgida* | 9561-AMPH015 | MG027260 | MG027424 | Stepanek & Kociolek (2019) |
| *Halamphora sydowii* | 6808-AMPH028 | MG027265 | MG027429 | Stepanek & Kociolek (2019) |
| *Halamphora tenuicostata* | 6863-AMPH042 | MG027271 | MG027435 | Stepanek & Kociolek (2019) |
| *Halamphora tenuis* | 6823-AMPH034 | MG027269 | MG027433 | Stepanek & Kociolek (2019) |
| *Halamphora tumida* | 8880-AMPH149 | MG027319 | MG027485 | Stepanek & Kociolek (2019) |
| *Halamphora turgida* var. *lacustris* | 10126-AMPH192 | MG027336 | MG027502 | Stepanek & Kociolek (2019) |
| *Halamphora veneta* | 6020-AMPH005 | KJ463452 | KJ463482 | Stepanek & Kociolek (2014) |
| *Tetramphora chilensis* | 8531-Amph132 | KU665638 | KU665639 | Stepanek & Kociolek (2016) |
